# Morphology and Phylogeny Reveal New Species and Records of *Diplodia*, *Dothiorella*, and *Phaeobotryon* Associated with Tree Cankers in Xizang, China

**DOI:** 10.3390/jof11050331

**Published:** 2025-04-22

**Authors:** Jia Zhou, Aining Li, Ning Jiang

**Affiliations:** 1 Beijing Key Laboratory for Forest Pest Control, Beijing Forestry University, Beijing 100083, China; z_houjia@bjfu.edu.cn; 2 Key Laboratory of Biodiversity Conservation of National Forestry and Grassland Administration, Ecology and Nature Conservation Institute, Chinese Academy of Forestry, Beijing 100091, China

**Keywords:** botryosphaeriaceae, botryosphaeriales, systematics, taxonomy

## Abstract

The fungal family Botryosphaeriaceae, which includes genera such as *Diplodia*, *Dothiorella*, and *Phaeobotryon*, comprises species commonly associated with woody plants such as endophytes, pathogens, and saprophytes. The Xizang Autonomous Region of China, known for its rich forest resources, harbors significant fungal diversity. However, limited research has been conducted on plant-disease-associated fungi in this region. In this study, we employed morphological characteristics and molecular phylogenetic analyses of the internal transcribed spacer region of rDNA (ITS), the ribosomal large subunit (LSU), the translation elongation factor 1-alpha (*tef1*) gene, and the partial beta-tubulin (*tub2*) gene to identify fungal species. As a result, two new species, *Diplodia salicicola* sp. nov. and *Phaeobotryon xizangense* sp. nov., are proposed and described herein. Additionally, *Di*. *corticola*, *Di*. *mutila*, *Do*. *acericola*, *Do*. *magnoliae*, *Do*. *vidmadera*, *Do*. *yunnana* comb. nov., and *Do*. *zanthoxyli* are reported for the first time in Xizang. Our findings contribute to advancing the knowledge of fungal biodiversity in Xizang’s high-altitude ecosystems.

## 1. Introduction

Fungi play a significant role in forest ecosystems and are closely associated with forest health, often causing various diseases including canker, leaf spots, fruit rot, and other ailments [1,2,3,4,5,6,7,8]. Members of the Botryosphaeriales usually inhabit tree hosts, existing as endophytes, saprophytes, or pathogens [9,10,11,12,13,14]. For instance, *Aplosporella prunicola*, *Botryosphaeria dothidea*, and *Phyllosticta capitalensis* are known to cause leaf spots on *Castanea mollissima* [15]; *Melanops chinensis* is linked to oak canker disease [16]; and *Lasiodiplodia cinnamomi* is responsible for branch canker in *Cinnamomum camphora* [17].

The fungal order Botryosphaeriales was initially established to encompass a single family, Botryosphaeriaceae [18]. However, recent studies have significantly expanded our understanding of this order, leading to the discovery of new families, genera, and species [19,20,21,22,23,24]. Currently, six phylogenetically distinct lineages, representing six families, are widely recognized within Botryosphaeriales: Aplosporellaceae, Botryosphaeriaceae, Melanopsaceae, Phyllostictaceae, Planistromellaceae, and Saccharataceae [21,24]. Among these, Botryosphaeriaceae is the most diverse, comprising the majority of genera and species, whereas Melanopsaceae is the least diverse, containing only a single genus with a limited number of species [16,25,26,27,28,29,30,31].

*Diplodia* is known to produce two distinct types of conidia. In type 1, the conidia are initially hyaline and aseptate but gradually transition to pale or dark brown and develop one or several septa as they mature. In contrast, conidia of type 2 become pigmented early in their development and rarely form septa [22,24,29]. Due to the overlapping morphological characteristics among species, molecular phylogenetic analysis is essential for accurate species identification within this genus [32,33,34].

*Dothiorella* has undergone significant conceptual changes over time [29]. Currently, it is recognized as a distinct lineage within the family Botryosphaeriaceae [19,20,21,29,35,36]. Species identification of *Dothiorella* and the other botryosphaeriaceous genera primarily relies on anamorphic and cultural characteristics, supplemented by molecular phylogenetic analysis, due to the rarity of their teleomorphic stages [35,36,37,38,39].

*Phaeobotryon* was established to accommodate the species previously known as *Dothidea cercidis*, now classified as *Phaeobotryon cercidis* [40]. This genus is characterized by its distinctive 2-septate, brown ascospores, which exhibit conical, apiculate-elliptic to oblong or obovoid shapes at both ends, along with hyaline or brown conidia [29,41,42,43,44,45]. Recently, several additional species have been incorporated into this genus based on a combination of morphological characteristics and molecular phylogenetic evidence [41,42,43,44,45].

In the present study, symptoms of Botryosphaeriaceae-associated canker were observed on various tree hosts in Xizang, China. The objectives of this study were to identify the fungi from the diseased branches and to describe and characterize new species using a combination of molecular and morphological approaches.

## 2. Materials and Methods

### 2.1. Specimens and Strains

In the summer of 2024, field investigations were carried out in the southeastern regions of Xizang to collect fungi from virgin forests. During the sampling process, tree branches and twigs exhibiting conspicuous fungal fruiting bodies were carefully selected. These branches were then cut into 15 cm segments, systematically packaged in paper bags, and subsequently transported to the laboratory for comprehensive analysis and further research.

Using sterile surgical blades, fungal fruiting bodies were carefully dissected, and spore masses were aseptically transferred onto the surface of potato dextrose agar (PDA) medium (containing 200 g potato infusion, 20 g glucose, 20 g agar, and distilled water to make 1000 mL final volume) with sterilized inoculation needles. The inoculated Petri dishes were maintained at 25 °C under complete darkness to facilitate spore germination. Voucher specimens were deposited in the Herbarium of the Chinese Academy of Forestry (CAF), while the pure cultures were preserved in the China Forestry Culture Collection Center (CFCC) for long-term storage and future reference.

### 2.2. Morphological Observations

Comprehensive morphological characterization was performed based on the examination of naturally developed fungal conidiomata on host twigs and branches. The fruiting bodies were meticulously sectioned using sterile surgical blades and subsequently documented using a Zeiss Discovery V8 stereomicroscope (Carl Zeiss AG, Jena, Germany) equipped with a digital imaging system. Detailed microscopic examination was conducted using an Olympus BX51 compound microscope (Olympus Corporation, Tokyo, Japan) equipped with a camera Axiocam 208 color and and ZEN lite Software (https://www.zeiss.com/microscopy/en/products/software/zeiss-zen-lite.html, accessed on 5 August 2024) to analyze and photograph critical taxonomic features, including conidiophores, conidiogenous cells, and conidia. For quantitative analysis, fifty conidia were randomly selected and measured to determine their dimensional characteristics.

### 2.3. DNA Extraction and Amplification

Genomic DNA was extracted from fungal colonies cultivated on PDA plates using the cetyltrimethylammonium bromide (CTAB) method [46]. For isolates belonging to *Diplodia* and *Dothiorella*, three genomic regions were amplified: the internal transcribed spacer region of ribosomal DNA (ITS), the translation elongation factor 1-alpha (*tef1*) gene, and the partial beta-tubulin (*tub2*) gene. For *Phaeobotryon* isolates, the ITS region, the large subunit ribosomal RNA gene (LSU), and the *tef1* gene were targeted. PCR amplification was performed using the following primer pairs: ITS1/ITS4 for ITS, LR0R/LR5 for LSU, EF1-728F/EF1-986R for *tef1*, and Bt2a/Bt2b for *tub2* [47,48,49,50]. The thermal cycling conditions consisted of an initial denaturation at 94 °C for 5 min, followed by 35 cycles of denaturation at 94 °C for 30 s, annealing at 52 °C (for ITS and LSU) or 54 °C (for *tef1* and *tub2*) for 50 s, and extension at 72 °C for 1 min, with a final elongation step at 72 °C for 7 min. Purified PCR products were sequenced bidirectionally by Sangon Biotech Co., Ltd. (Beijing, China).

### 2.4. Molecular Phylogeny

The obtained sequences were initially identified through BLASTn searches against the NCBI GenBank database to verify their taxonomic classification. Reference sequences of *Diplodia*, *Dothiorella*, and *Phaeobotryon* were retrieved from recent publications (Table 1, Table 2 and Table 3) and downloaded from GenBank. For each genus, individual loci (ITS, LSU, *tef1*, and *tub2*) were aligned using MAFFT v. 6.0 and manually refined in MEGA v. 6.0. [51,52]. Subsequently, concatenated datasets were prepared: ITS, *tef1*, and *tub2* for *Diplodia* and *Dothiorella*, and ITS, LSU, and *tef1* for *Phaeobotryon*. Phylogenetic analyses were conducted using both maximum likelihood (ML) and Bayesian inference (BI) approaches through the CIPRES Science Gateway platform [53,54,55]. The general time reversible (GTR) model with gamma-distributed rate variation was selected as the optimal substitution model. For ML analysis, nodal support was assessed with 1000 bootstrap replicates. Bayesian analysis was performed with four independent Markov Chain Monte Carlo (MCMC) runs of 1,000,000 generations each, sampling every 1000 generations. The first 25% of trees were discarded as burn-in, and the remaining trees were used to construct a 50% majority-rule consensus tree. Phylogenetic trees were visualized and annotated using FigTree v. 1.4.4, with final graphical editing performed in Adobe Illustrator 2020.

## 3. Results

### 3.1. Phylogeny

**Figure 1:**
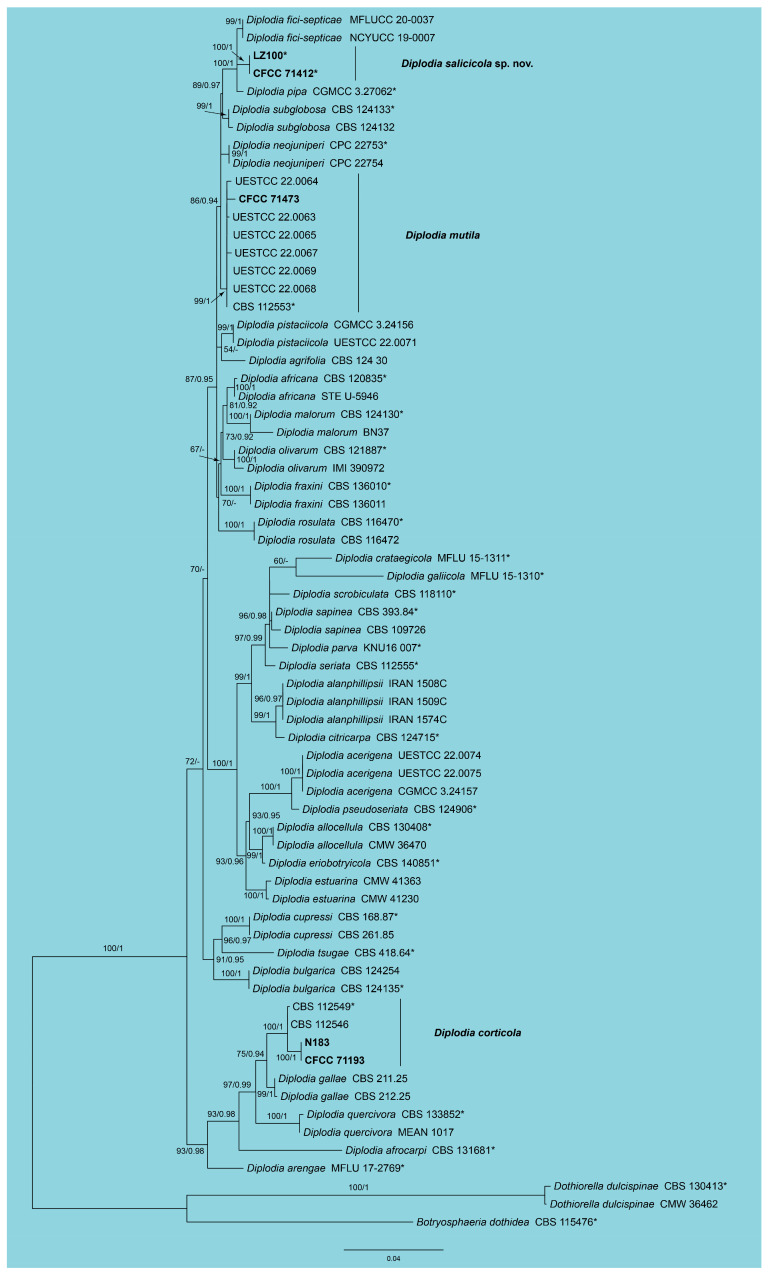
Phylogram of *Diplodia* resulting from a maximum likelihood analysis based on the combined dataset of ITS, *tef1*, and *tub2*. Numbers above the branches indicate ML bootstraps (left, ML BS ≥ 50%) and Bayesian Posterior Probabilities (right, BPP ≥ 0.90). The tree is rooted with *Botryosphaeria dothidea* (CBS 115476) and *Dothiorella dulcispinae* (CBS 130413 and CMW 36462). The ex-type strains are indicated by an asterisk (*), and strains from the present study are in black bold.

In the phylogenetic analysis of *Diplodia*, a combined dataset comprising ITS, *tef1*, and *tub2* sequences from 68 strains was utilized. The final alignment spanned 1302 characters, distributed as follows: 568 characters from ITS, 303 from *tef1*, and 431 from *tub2*. The maximum likelihood (ML) optimization of the best RAxML tree yielded a likelihood value of −6096.16. The alignment matrix contained 540 distinct patterns, with 13.49% of the characters being undetermined or gaps. The estimated base frequencies were A = 0.204491, C = 0.308989, G = 0.259443, and T = 0.227077. The substitution rates were AC = 0.839353, AG = 2.834662, AT = 0.813846, CG = 0.921389, CT = 4.425100, and GT = 1.0. The gamma distribution shape parameter (α) was 0.257468. The topology of the phylogenetic tree constructed in this study was highly consistent with those reported in previous publications. Both RAxML and Bayesian analyses produced congruent topologies for the isolates examined. Specifically, isolates CFCC 71412 and LZ100 formed a distinct clade closely related to *Diplodia fici*-*septicae* and *D*. *pipa*, supported by high bootstrap (BS = 100) and Bayesian posterior probability (BPP = 1) values. Isolate CFCC 71471 clustered with strains of *D*. *mutila*, while isolates CFCC 71193 and N183 formed a clade with strains of *D*. *corticola* (Figure 1). Based on these findings, five *Diplodia* isolates from this study were identified as *D*. *salicicola* sp. nov., *D*. *mutila*, and *D*. *corticola*.

**Figure 2:**
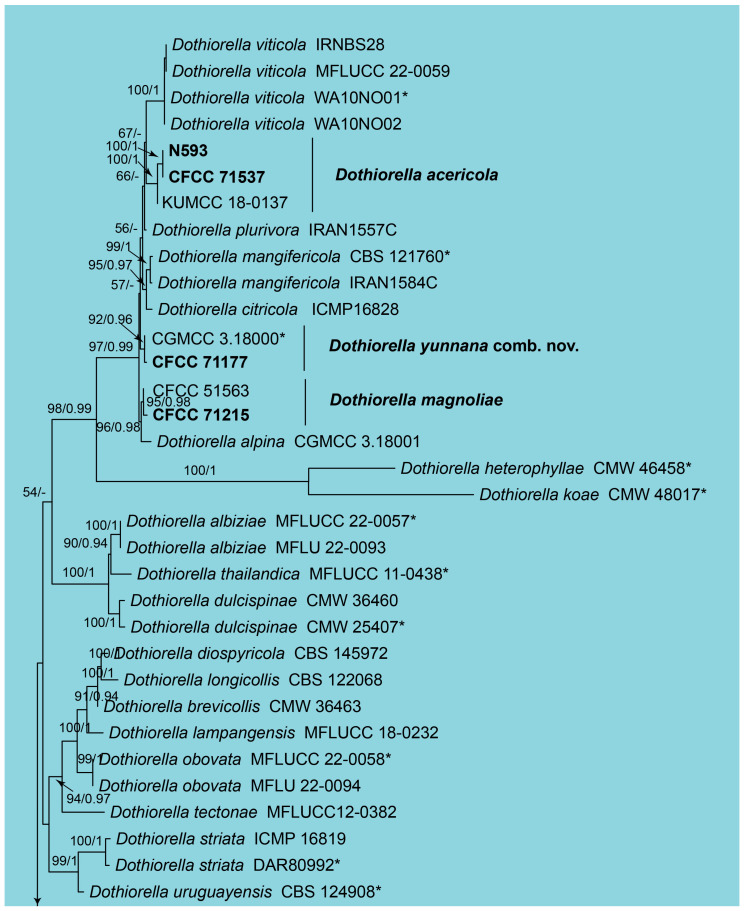

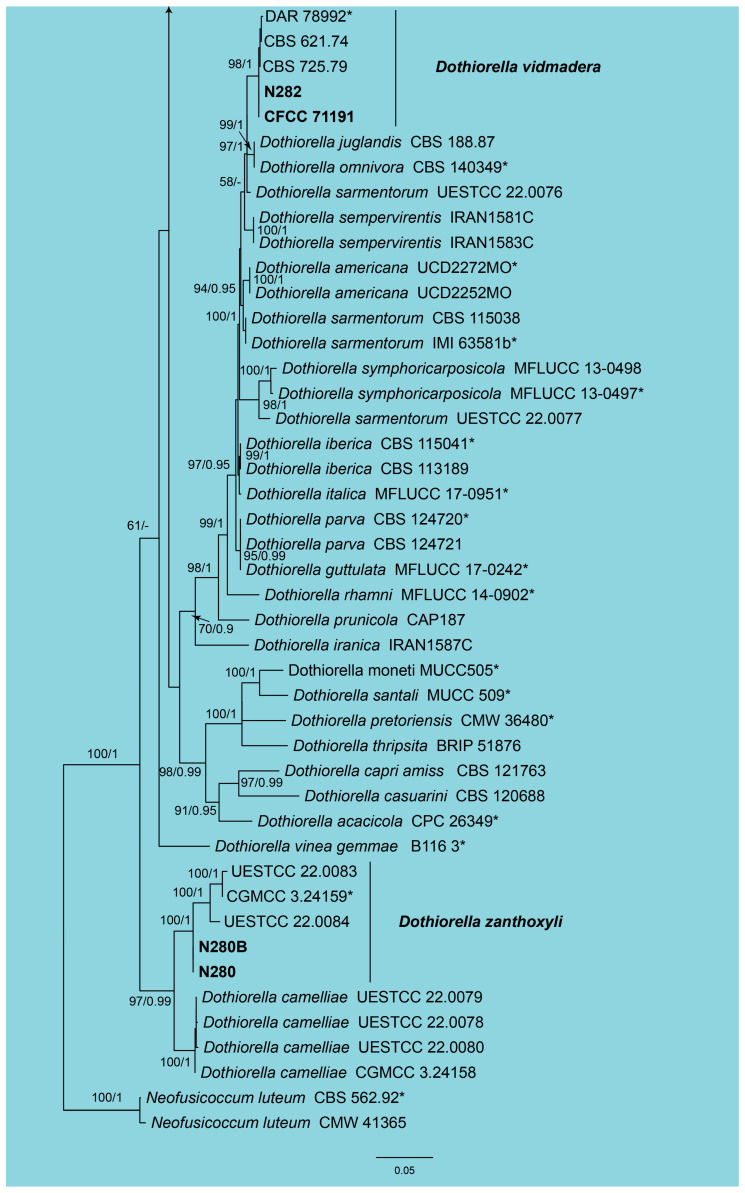
Phylogram of *Dothiorella* resulting from a maximum likelihood analysis based on the combined dataset of ITS, *tef1*, and *tub2*. Numbers above the branches indicate ML bootstraps (left, ML BS ≥ 50%) and Bayesian Posterior Probabilities (right, BPP ≥ 0.90). The tree is rooted with *Neofusicoccum luteum* (CBS 562.92 and CMW 41365). The ex-type strains are indicated by an asterisk (*), and strains from the present study are in black bold.

In the phylogenetic analysis of *Dothiorella*, a combined dataset comprising ITS, *tef1*, and *tub2* sequences from 78 strains was utilized. The final alignment spanned 1384 characters, distributed as follows: 661 characters from ITS, 294 from *tef1*, and 429 from *tub2*. The maximum likelihood (ML) optimization of the best RAxML tree yielded a likelihood value of −8608.95. The alignment matrix contained 661 distinct patterns, with 24.09% of the characters being undetermined or gaps. The estimated base frequencies were A = 0.210908, C = 0.305139, G = 0.251287, and T = 0.232666. The substitution rates were AC = 0.875931, AG = 2.225085, AT = 1.052270, CG = 0.978866, CT = 3.644353, and GT = 1.0. The gamma distribution shape parameter (α) was 0.250867. The topology of the phylogenetic tree constructed in this study was consistent with those reported in previous publications. Both RAxML and Bayesian analyses produced congruent topologies for the isolates examined. Based on the phylogeny (Figure 2), eight *Dothiorella* isolates from this study were identified as *Do*. *acericola*, *Do*. *yunnana*, *Do*. *magnoliae*, *Do*. *Vidmadera*, and *Do*. *zanthoxyli*.

**Figure 3:**
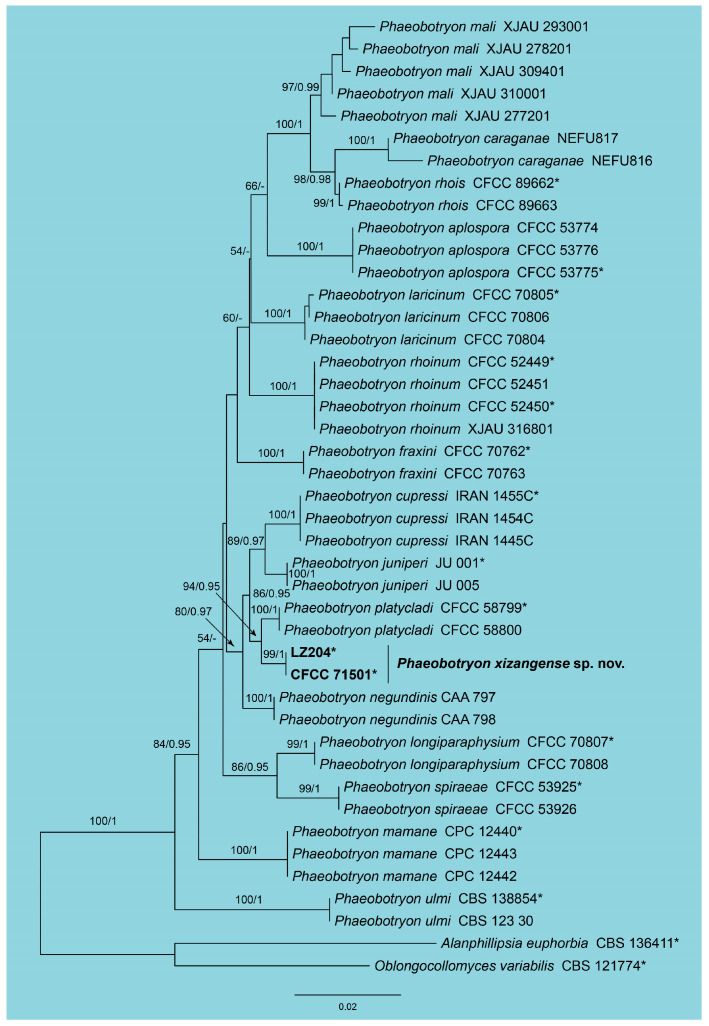
Phylogram of *Phaeobotryon* resulting from a maximum likelihood analysis based on the combined dataset of ITS, *tef1*, and *tub2*. Numbers above the branches indicate ML bootstraps (left, ML BS ≥ 50%) and Bayesian Posterior Probabilities (right, BPP ≥ 0.90). The tree is rooted with *Alanphillipsia euphorbia* (CBS 136411) and *Oblongocollomyces variabilis* (CBS 121774). The ex-type strains are indicated by an asterisk (*), and strains from the present study are in black bold.

In the phylogenetic analysis of *Phaeobotryon*, a combined dataset comprising ITS, LSU, and *tef1* sequences from 43 strains was utilized. The final alignment spanned 1891 characters, distributed as follows: 559 characters from ITS, 785 from LSU, and 547 from *tef1*. The maximum likelihood (ML) optimization of the best RAxML tree yielded a likelihood value of −5235.17. The alignment matrix contained 379 distinct patterns, with 18.78% of the characters being undetermined or gaps. The estimated base frequencies were A = 0.232859, C = 0.255406, G = 0.284479, and T = 0.227256. The substitution rates were AC = 0.942249, AG = 3.001570, AT = 0.626571, CG = 1.509093, CT = 5.496302, and GT = 1.0. The gamma distribution shape parameter (α) was 0.257468. The topology of the phylogenetic tree constructed in this study was highly consistent with those reported in previous publications. Both RAxML and Bayesian analyses produced congruent topologies for the isolates examined. Two isolates from the present study clustered into a separate clade from the other known species, named as *Phaeobotryon xizangense* sp. nov (Figure 3).

### 3.2. Taxonomy

***Diplodia corticola*** A.J.L. Phillips, A. Alves & J. Luque, in Alves, Correia, Luque & Phillips, Mycologia 96(3): 603 (2004)

Materials examined: CHINA, Xizang Autonomous Region, Linzhi City, Gongbujiangda County, Gongbujiangda Town, Apeixin Village, on cankered twigs and branches of *Prunus mira*, 7 July 2024, Ning Jiang, Jiangrong Li, Jieting Li & Liangna Guo (cultures CFCC 71193, N183).

Notes: *Diplodia corticola* is recognized as a pathogen affecting *Quercus* spp. in Europe and the USA, and it has also been reported to inhabit *Eucalyptus globulus* and *Pinus pinaster* [32,37]. In this study, two isolates obtained from *Prunus mira* in China were identified as *D*. *corticola* based on molecular evidence (Figure 1). This finding expands the known host range and geographic distribution of *D*. *corticola*.

***Diplodia mutila*** (Fr.) Fr., Annls Sci. Nat., Bot., sér. 2 1: 349 (1834)

Materials examined: CHINA, Xizang Autonomous Region, Linzhi City, Bomi County, on cankered branches of *Prunus armeniaca*, 24 October 2024, Ning Jiang, Min Liu, Jieting Li & Yi Li (culture CFCC 71473).

Notes: *Diplodia mutila* is associated with a diverse range of woody hosts globally, including species of *Malus*, *Quercus*, *Vitis*, and others [31,33]. In this study, we isolated a new strain of *D. mutila* from *Prunus armeniaca*, which were confidently identified through molecular data (Figure 1).

***Diplodia*** ***salicicola*** Ning Jiang, sp. nov.

Figure 4

MycoBank: MB858270

Etymology: Named after the host genus, *Salix* and “*cola*” = inhabiting.

Description: *Conidiomata* pycnidial, scattered, subglobose to globose, semi-immersed to erumpent, unilocular, 250–450 μm diam. *Disc* brown to black, 100–200 μm in diam. *Ostioles* single, central, papillate, 20–45 μm. *Paraphyses* present, hyaline, thin-walled, arising from the conidiogenous layer, extending above the level of developing conidia, tip rounded, aseptate, up to 72 × 2 μm. *Conidiophores* reduced to conidiogenous cells. *Conidiogenous cells* hyaline, smooth, thin-walled, cylindrical, holoblastic, phialidic, proliferating internally with visible periclinal thickening, (9.5–)11.5–25.5(–37) × (2.5–)3–4.5(–6) μm. *Conidia* initially hyaline, becoming brown with age, oval to cylindrical, smooth with granular contents, guttulate, both ends broadly rounded, aseptate, (26.5–)27.5–29(–29.5) × (10–)11–13(–13.5) μm.

Culture characteristics: *Colonies* on PDA flat, spreading, with flocculent mycelium and even edges, initially white to grey, becoming brown after 10 d, reaching a 90 mm diameter after 10 days at 25 °C in the dark.

Materials examined: CHINA, Xizang Autonomous Region, Linzhi City, Chayu County, on cankered twigs and branches of *Salix takasagoalpina*, 22 October 2024, Ning Jiang, Min Liu, Jieting Li & Yi Li (holotype CAF800143); ex-type cultures CFCC 71412, LZ100.

Notes: *Diplodia salicicola*, isolated from *Salix takasagoalpina* in Xizang, is phylogenetically closely related to *D*. *fici-septicae* from *Ficus septica* in Taiwan and *D*. *pipa* from *Eriobotrya japonica* in Yunnan (Figure 1). Although all three species were discovered in China, they exhibit distinct morphological and molecular characteristics. Morphologically, *D*. *salicicola* can be distinguished from *D*. *fici-septicae* by its longer conidiogenous cells (11.5–25.5 × 3–4.5 μm in *D*. *salicicola* vs. 4–7 × 3–5 μm in *D*. *fici-septicae*), and from *D*. *pipa* by its aseptate mature conidia. Additionally, *D. salicicola* differs from *D*. *fici-septicae* and *D*. *pipa* at the molecular level, with sequence differences of 1/545 bp in ITS and 18/315 bp in *tef1* compared to *D*. *fici-septicae* and 4/545 bp in ITS, 16/315 bp in *tef1*, and 2/404 bp in *tub2* compared to *D*. *pipa* [34,43].

**Figure 4:**
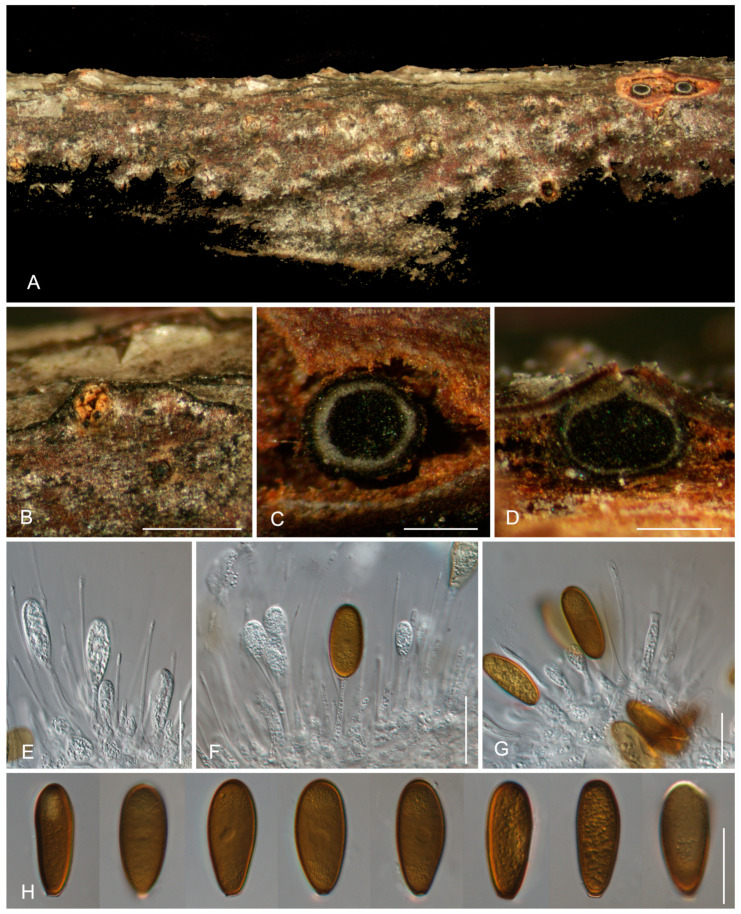
Morphology of *Diplodia salicicola* from *Salix takasagoalpina.* (**A**,**B**) Conidiomata formed on branches. (**C**) Transverse section through a conidioma. (**D**) Longitudinal section of a conidioma. (**E**–**G**) Conidiogenous cells with attached conidia. (**H**) Conidia. Scale bars: (**B**) = 500 μm; (**C**,**D**) = 300 μm; (**E**–**H**) = 20 μm.

***Dothiorella acericola*** Phookamsak, Tennakoon & K.D. Hyde, Fungal Diversity 95: 78 (2019)

Materials examined: CHINA, Xizang Autonomous Region, Linzhi City, Bomi County, Yigong Town, on dead branches of an unknown host, 25 October 2024, Ning Jiang, Min Liu, Jieting Li & Yi Li (cultures CFCC 71537, N593).

Notes: *Dothiorella acericola* was first identified on dead hanging twigs of *Acer palmatum* in Yunnan Province, China, and was later reported to be associated with branch cankers of *Ziziphus jujuba* in Beijing [56,57]. In the present study, we report, for the first time, the occurrence of this fungal species on dead branches in Xizang Autonomous Region, thereby expanding its known geographical distribution.

***Dothiorella magnoliae*** C.M. Tian & C.J. You, Mycosphere 8(2): 1035 (2017)

Materials examined: CHINA, Xizang Autonomous Region, Linzhi City, Bayi District, on cankered branches of *Albizia julibrissin*, 8 July 2024, Ning Jiang, Jieting Li, Yi Li & Ji Qiang (culture CFCC 71215).

Notes: *Dothiorella magnoliae* was originally described based on two strains isolated from Magnolia grandiflora in Sichuan Province, China [39]. In the present study, we report the first isolation of this fungal species from *Albizia julibrissin*, representing a new host record for this pathogen.

***Dothiorella vidmadera*** W.M. Pitt, Úrbez-Torr. & Trouillas, Fungal Diversity 61(1): 216 (2013)

Materials examined: CHINA, Xizang Autonomous Region, Linzhi City, Bomi County, Yigong Town, on cankered branches of *Chaenomeles cathayensis*, 10 July 2024, Ning Jiang, Jieting Li & Haoyin Zhang (cultures CFCC 71191, N282).

Notes: *Dothiorella vidmadera* was originally reported as a pathogen associated with grapevines (Vitis vinifera) in Australia [38]. In the present study, we identified two strains isolated from *Chaenomeles cathayensis* in Xizang Autonomous Region, China, as *D. vidmadera*, representing both a new host association and a significant expansion of its known geographical distribution.

***Dothiorella yunnana*** (Y. Zhang ter & Min Zhang) Ning Jiang, comb. nov.

MycoBank: MB858272

Basionym: *Spencermartinsia yunnana* Y. Zhang ter & Min Zhang, Mycosphere 7(7): 1060 (2016)

Materials examined: CHINA, Xizang Autonomous Region, Linzhi City, Lang County, on cankered branches of *Rosa chinensis*, 5 July 2024, Ning Jiang, Jiangrong Li, Jieting Li & Liangna Guo (culture CFCC 71177).

Notes: The genus *Dothiorella* was resurrected to accommodate species characterized by conidia that become pigmented while still attached to their conidiogenous cells [30]. Concurrently, the genus *Spencermartinsia* was established to include *Dothiorella*-like species exhibiting apiculate ascospores [58]. However, molecular phylogenetic evidence has demonstrated that the presence of apiculate ascospores within the Botryosphaeriales is not a reliable taxonomic character for generic delineation [59]. *Spencermartinsia yunnana* was originally described from multiple hosts, including *Acer buergerianum*, *Camellia* sp., *Poncirus trifoliata*, and *Ternstroemia gymnanthera*, with its taxonomic status validated through the designation of a holotype [36,60]. In the present study, based on comprehensive phylogenetic analyses, we further confirm that *Spencermartinsia* should be treated as a synonym of Dothiorella. Consequently, *Spencermartinsia yunnana* is formally transferred to *Dothiorella* as *Do. yunnana*.

***Dothiorella zanthoxyli*** L.W. Li & Jian K. Liu, MycoKeys 97: 98 (2023)

Materials examined: CHINA, Xizang Autonomous Region, Linzhi City, Bomi County, Yigong Town, on cankered branches of *Diospyros lotus*, 10 July 2024, Ning Jiang, Jieting Li & Haoyin Zhang (cultures CFCC 71191, N282).

Notes: *Dothiorella zanthoxyli* was recently described as a species isolated from decaying branches of *Zanthoxylum bungeanum* in Sichuan, China [41]. In this study, we identified two strains obtained from cankered branches of Diospyros lotus in Xizang, China, as *Do. zanthoxyli* based on molecular phylogeny (Figure 2). This finding represents the first report of *Do. zanthoxyli* on *Diospyros lotus*, thereby establishing it as a new host for this fungal species.

***Phaeobotryon xizangense*** Ning Jiang, sp. nov.

Figure 5

MycoBank: MB858271

Etymology: Named after the collection site of the holotype, Xizang Autonomous Region.

Description: *Conidiomata* pycnidial, scattered, subglobose to globose, semi-immersed to erumpent, unilocular, and 400–650 μm diam. *Disc* black, 200–400 μm in diam. *Ostioles* single, central, papillate, and 85–190 μm. *Paraphyses* present, hyaline, thin-walled, arising from the conidiogenous layer, extending above the level of developing conidia, tip rounded, aseptate, and up to 58 × 3.5 μm. *Conidiophores* reduced to conidiogenous cells. *Conidiogenous cells* hyaline, smooth, thin-walled, cylindrical, holoblastic, phialidic, and proliferating internally with visible periclinal thickening, (10–)12.5–21.5(–25) × (2.5–)3–4.5(–5.5) μm. *Conidia* initially hyaline, becoming brown with age, oval to cylindrical, smooth with granular contents, guttulate, both ends broadly rounded, initially aseptate, and becoming 1-septate, (25–)26.5–30(–32.5) × (11.5–)13–15.5(–16) μm.

Culture characteristics: *Colonies* on PDA flat, spreading, with flocculent mycelium and even edges, initially white, becoming brown to black after 10 d, and reaching a 90 mm diameter after 10 days at 25 °C in the dark.

Materials examined: CHINA, Xizang Autonomous Region, Linzhi City, Bomi County, Yigong Town, on cankered twigs and branches of *Platycladus orientalis*, 25 October 2024, Ning Jiang, Min Liu, Jieting Li & Yi Li (holotype CAF800144); ex-type cultures CFCC 71501, LZ204.

Notes: In the phylogram (Figure 3), *Phaeobotryon xizangense* from Xizang, China, clusters closely with *P. platycladi* from Beijing, China. Despite both species sharing the same host, *Platycladus orientalis*, they can be readily distinguished by their conidial dimensions: *P*. *xizangense* exhibits conidia measuring 26.5–30 × 13–15.5 μm, whereas *P*. *platycladi* has conidia of a different size 23–31 × 9.5–12.5 μm. In addition, *P*. *xizangense* differs from *P*. *platycladi* in sequence data (4/463 bp in ITS, 1/556 bp in LSU, 22/312 bp in *tef1*) [42].

**Figure 5:**
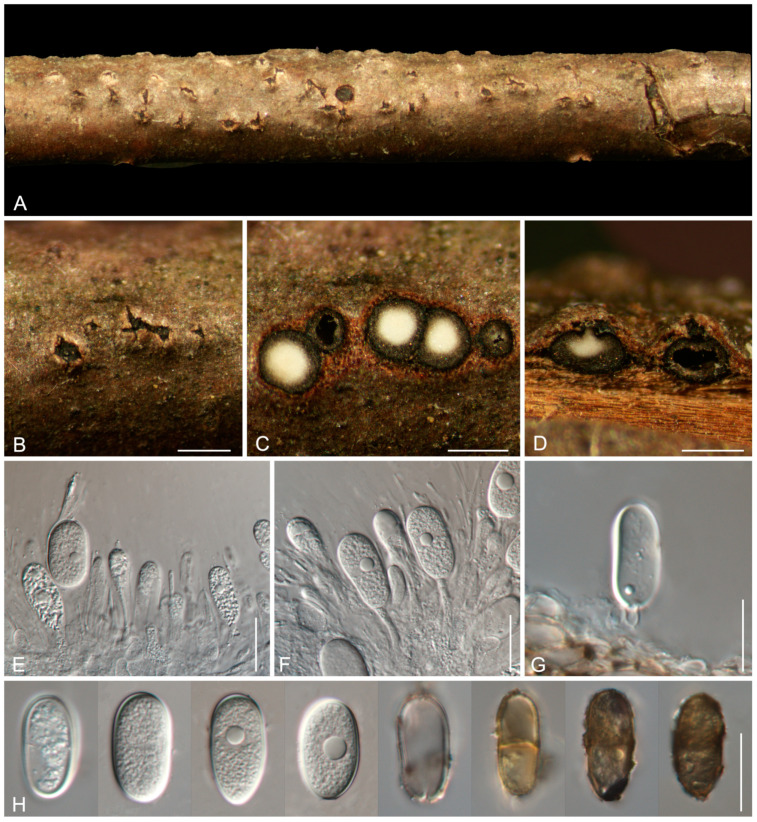
Morphology of *Phaeobotryon xizangense* from *Platycladus orientalis.* (**A**,**B**) Conidiomata formed on branches. (**C**) Transverse section through the conidiomata. (**D**) Longitudinal section of the conidiomata. (**E**–**G**) Conidiogenous cells with attached conidia. (**H**) Conidia. Scale bars: (**B**–**D**) = 500 μm; (**E**–**H**) = 20 μm.

## 4. Discussion

In this study, species belonging to the genera *Diplodia*, *Dothiorella*, and *Phaeobotryon* were investigated in Xizang, a region known for its rich fungal diversity but which is largely understudied. Using an integrative taxonomic approach that combines morphological characterization and molecular phylogeny, several species were identified, including *Diplodia corticola*, *Di. mutila*, *Di. salicicola* sp. nov., *Do. acericola*, *Do. magnoliae*, *Do. vidmadera*, *Do. yunnana* comb. nov., *Do. zanthoxyli*, and *Phaeobotryon xizangense* sp. nov., isolated from various tree hosts. These findings substantially advance our understanding of fungal species diversity of Botryosphaeriales and their host associations and distribution patterns.

Members of Botryosphaeriales are widely recognized as plant pathogens, yet the ecological roles of many species remain unconfirmed due to a lack of pathogenicity testing [61,62,63]. A similar situation arises in this study, where most identified species were found in association with canker symptoms. However, due to insufficient host materials, conducting rigorous pathogenicity tests proved challenging. Given the ecological and economic significance of botryosphaerialean fungi, increased funding and research attention should be directed toward these fungi and their associated diseases. Such efforts would not only advance fungal taxonomy but also contribute to the understanding and management of forest health.

Generic boundaries within Botryosphaeriales are often morphologically ambiguous [14,19,20,21]. A notable example is the previous placement of several *Dothiorella* species into *Spencermartinsia* based on the presence of apiculate ascospores [30,58,59]. However, this characteristic was later determined to be unreliable for genus delineation, leading to the synonymization of *Spencermartinsia* with *Dothiorella*, as supported by multigene phylogenetic analyses [36,59,60]. In this study, we reclassify *Spencermartinsia yunnana* as *Dothiorella yunnana* to clarify the taxonomic boundaries within the genus *Dothiorella*.

Septation and pigmentation are not consistently reliable diagnostic features within Botryosphaeriales [29,64]. For instance, in the genus *Diplodia*, some species, such as *Di. salicicola*, produce aseptate and pigmented conidia, while others, like *Di*. *afrocarpi*, initially form hyaline and aseptate conidia that later transition to dark brown and develop a septum as they mature [64]. Despite this variability in septation and pigmentation, these species form a strongly supported monophyletic lineage within Botryosphaeriales [21,24]. Therefore, future studies incorporating broader collections and additional data are essential to refine the classification of genera within Botryosphaeriaceae and to establish more robust taxonomic boundaries.

## Figures and Tables

**Table 1:** Isolates and GenBank accession numbers used in the genus *Diplodia*.

Species	Isolates	GenBank Accession Number		
		ITS	*tef1*	*tub2*
*Botryosphaeria dothidea*	CBS 115476 *	AY236949	AY236898	AY236927
*Diplodia acerigena*	CGMCC 3.24157	OQ190518	OQ241452	NA
*Diplodia acerigena*	UESTCC 22.0074	OQ190519	OQ241453	OQ338163
*Diplodia acerigena*	UESTCC 22.0075	OQ190520	OQ241454	OQ338164
*Diplodia africana*	CBS 120835 *	KF766155	KF766397	KF766129
*Diplodia africana*	STE-U 5946	EF445344	EF445383	NA
*Diplodia afrocarpi*	CBS 131681 *	MT587333	MT592035	MT592471
*Diplodia agrifolia*	CBS 124.30	KX464087	KX464557	KX464783
*Diplodia alanphillipsii*	IRAN 1508C	KF890208	KF890190	NA
*Diplodia alanphillipsii*	IRAN 1509C	KF890209	KF890191	NA
*Diplodia alanphillipsii*	IRAN 1574C	MT258875	MT270153	NA
*Diplodia allocellula*	CBS 130408 *	JQ239397	JQ239384	JQ239378
*Diplodia allocellula*	CMW 36470	JQ239399	JQ239386	JQ239380
*Diplodia arengae*	MFLU 17-2769 *	MG762771	MG762774	MG783039
*Diplodia bulgarica*	CBS 124254	GQ923853	GQ923821	NA
*Diplodia bulgarica*	CBS 124135 *	GQ923852	GQ923820	NA
*Diplodia citricarpa*	CBS 124715 *	KF890207	KF890189	KX464784
*Diplodia corticola*	CBS 112549 *	AY259100	AY573227	DQ458853
*Diplodia corticola*	CBS 112546	AY259090	EU673310	EU673117
***Diplodia corticola***	**CFCC 71193**	**PV264850**	**NA**	**PV339814**
***Diplodia corticola***	**N183**	**PV264851**	**NA**	**PV339815**
*Diplodia crataegicola*	MFLU 15-1311 *	KT290244	KT290248	KT290246
*Diplodia cupressi*	CBS 168.87 *	DQ458893	DQ458878	DQ458861
*Diplodia cupressi*	CBS 261.85	DQ458894	DQ458879	DQ458862
*Diplodia eriobotryicola*	CBS 140851 *	KT240355	KT240193	MG015806
*Diplodia estuarina*	CMW 41363	KP860829	KP860674	KP860752
*Diplodia estuarina*	CMW 41230	KP860830	KP860675	KP860753
*Diplodia fici-septicae*	MFLUCC 20-0037	MW063180	MW183802	NA
*Diplodia fici-septicae*	NCYUCC 19-0007	MW063181	MW183803	NA
*Diplodia fraxini*	CBS 136010 *	KF307700	KF318747	MG015807
*Diplodia fraxini*	CBS 136011	KF307711	KF318748	MG015808
*Diplodia galiicola*	MFLU15-1310 *	KT290245	KT290249	MT592471
*Diplodia gallae*	CBS 211.25	KX464090	KX464564	KX464795
*Diplodia gallae*	CBS 212.25	KX464091	KX464565	KX464796
*Diplodia malorum*	CBS 124130 *	GQ923865	GQ923833	NA
*Diplodia malorum*	BN-37	KT240360	KT240198	NA
*Diplodia mutila*	CBS 112553 *	AY259093	AY573219	KY554743
*Diplodia mutila*	UESTCC 22.0064	OQ190521	OQ241455	OQ338165
*Diplodia mutila*	UESTCC 22.0065	OQ190522	OQ241456	OQ338166
*Diplodia mutila*	UESTCC 22.0069	OQ190523	OQ241457	OQ338167
*Diplodia mutila*	UESTCC 22.0068	OQ190524	OQ241458	OQ338168
*Diplodia mutila*	UESTCC 22.0067	OQ190525	OQ241459	OQ338169
*Diplodia mutila*	UESTCC 22.0063	OQ190526	OQ241460	OQ338170
***Diplodia mutila***	**CFCC 71473**	**PV264852**	**PV268109**	**PV339816**
*Diplodia neojuniperi*	CPC 22753 *	KM006431	KM006462	NA
*Diplodia neojuniperi*	CPC 22754	KM006432	KM006463	NA
*Diplodia olivarum*	CBS 121887 *	EU392302	EU392279	HQ660079
*Diplodia olivarum*	IMI 390972	HM028640	HQ660078	HQ660080
*Diplodia parva*	KNU16-007 *	LC417238	LC435495	LC522938
*Diplodia pipa*	CGMCC 3.27062 *	PP192032	PP197939	PP197952
*Diplodia pistaciicola*	CGMCC 3.24156	OQ190527	OQ241461	OQ338171
*Diplodia pistaciicola*	UESTCC 22.0071	OQ190528	OQ241462	OQ275062
*Diplodia pseudoseriata*	CBS 124906 *	EU080927	EU863181	MG015820
*Diplodia quercivora*	CBS 133852 *	JX894205	JX894229	MG015821
*Diplodia quercivora*	MEAN 1017	KU311198	KU311201	NA
*Diplodia rosulata*	CBS 116470 *	EU430265	EU430267	EU673132
*Diplodia rosulata*	CBS 116472	EU430266	EU430268	EU673131
***Diplodia salicicola* sp. nov.**	CFCC 71412 *	**PV264853**	**PV268110**	**PV339817**
***Diplodia salicicola* sp. nov.**	LZ100 *	**PV264854**	**PV268111**	**PV339818**
*Diplodia sapinea*	CBS 393.84 *	DQ458895	DQ458880	DQ458863
*Diplodia sapinea*	CBS 109726	KX464094	KX464568	KX464800
*Diplodia scrobiculata*	CBS 118110 *	AY253292	AY624253	AY624258
*Diplodia seriata*	CBS 112555 *	AY259094	AY573220	DQ458856
*Diplodia subglobosa*	CBS 124133 *	GQ923856	GQ923824	MT592576
*Diplodia subglobosa*	CBS 124132	DQ458887	DQ458871	DQ458852
*Diplodia tsugae*	CBS 418.64 *	DQ458888	DQ458873	DQ458855
*Dothiorella dulcispinae*	CBS 130413	JQ239400	JQ239387	JQ239373
*Dothiorella dulcispinae*	CMW 36462	JQ239402	JQ239389	JQ239375

Note: NA, not applicable. Ex-type strains are marked with *, and strains from present study are in black bold.

**Table 2:** Isolates and GenBank accession numbers used in the genus *Dothiorella*.

Species	Isolates	GenBank Accession Number		
		ITS	*tef1*	*tub2*
*Dothiorella acacicola*	CPC 26349 *	NR_145255	KX228376	NA
*Dothiorella acericola*	KUMCC 18-0137	MK359449	MK361182	NA
***Dothiorella acericola***	**CFCC 71537**	**PV264855**	**PV268112**	**PV339819**
***Dothiorella acericola***	**N593**	**PV264856**	**PV268113**	**PV339820**
*Dothiorella albiziae*	MFLUCC 22-0057 *	ON751762	ON799588	ON799590
*Dothiorella albiziae*	MFLU 22-0093	ON707683	NA	ON677453
*Dothiorella alpina*	CGMCC 3.18001	KX499645	KX499651	NA
*Dothiorella americana*	UCD2272MO *	HQ288219	HQ288263	HQ288298
*Dothiorella americana*	UCD2252MO	HQ288218	HQ288262	HQ288297
*Dothiorella brevicollis*	CMW 36463 *	NR_111703	JQ239390	JQ239371
*Dothiorella camelliae*	UESTCC 22.0080	OQ190530	NA	OQ275063
*Dothiorella camelliae*	UESTCC 22.0079	OQ190532	OQ241465	OQ275065
*Dothiorella camelliae*	UESTCC 22.0078	OQ190533	OQ241466	OQ275066
*Dothiorella camelliae*	CGMCC 3.24158	OQ190531	OQ241464	OQ275064
*Dothiorella capri-amiss*	CBS 121763	EU101323	EU101368	KX464850
*Dothiorella casuarini*	CBS 120688	DQ846773	DQ875331	NA
*Dothiorella chiangmaiensis*	YW177	NA	NA	NA
*Dothiorella citricola*	ICMP16828	EU673323	EU673290	EU673145
*Dothiorella diospyricola*	CBS 145972	MT587398	MT592110	MT592581
*Dothiorella dulcispinae*	CMW 36460	JQ239400	JQ239387	JQ239373
*Dothiorella dulcispinae*	CMW 25407 *	EU101300	MT592120	KX464862
*Dothiorella guttulata*	MFLUCC 17-0242 *	KY797637	NA	NA
*Dothiorella heterophyllae*	CMW 46458 *	MN103794	MH548348	MH548324
*Dothiorella iberica*	CBS 115041 *	AY573202	AY573222	EU673096
*Dothiorella iberica*	CBS 113189	AY573199	AY573230	KX464855
*Dothiorella iranica*	IRAN1587C	KC898231	KC898214	NA
*Dothiorella italica*	MFLUCC 17-0951 *	MG828897	MG829267	MT592592
*Dothiorella juglandis*	CBS 188.87	EU673316	EU673283	EU673119
*Dothiorella koae*	CMW 48017 *	MH447652	MH548338	MH548327
*Dothiorella lampangensis*	MFLUCC 18-0232	MK347758	MK340869	MK412874
*Dothiorella longicollis*	CBS 122068	EU144054	EU144069	NA
*Dothiorella magnoliae*	CFCC 51563 *	KY111247	KY213686	NA
***Dothiorella magnoliae***	**CFCC 71215**	**PV264857**	**NA**	**PV339821**
*Dothiorella mangifericola*	CBS 121760 *	EU101290	EU101335	KX464877
*Dothiorella mangifericola*	IRAN1584C	KC898221	KC898204	NA
*Dothiorella moneti*	MUCC505 *	EF591920	EF591971	EF591954
*Dothiorella obovata*	MFLUCC 22-0058 *	ON751763	ON799589	ON799591
*Dothiorella obovata*	MFLU 22-0094	ON707682	NA	ON677452
*Dothiorella omnivora*	CBS 140349 *	KP205497	KP205470	NA
*Dothiorella parva*	CBS 124720 *	KC898234	KC898217	KX464866
*Dothiorella parva*	CBS 124721	KX464123	KX464615	KX464867
*Dothiorella plurivora*	IRAN1557C	KC898225	KC898208	NA
*Dothiorella pretoriensis*	CMW 36480 *	JQ239405	JQ239392	JQ239376
*Dothiorella prunicola*	CAP187	EU673313	EU673280	EU673100
*Dothiorella rhamni*	MFLUCC 14-0902 *	KT240287	MT592111	MT592582
*Dothiorella santali*	MUCC 509 *	EF591924	EF591975	EF591958
*Dothiorella sarmentorum*	CBS 115038	AY573206	AY573223	EU673101
*Dothiorella sarmentorum*	IMI 63581b *	AY573212	AY573235	NA
*Dothiorella sarmentorum*	UESTCC 22.0076	OQ190534	NA	OQ275067
*Dothiorella sarmentorum*	UESTCC 22.0077	OQ190535	OQ241467	OQ275068
*Dothiorella sempervirentis*	IRAN1581C	KC898237	KC898219	KX464885
*Dothiorella sempervirentis*	IRAN1583C	KC898236	KC898220	KX464884
*Dothiorella striata*	ICMP 16819	EU673320	EU673287	EU673142
*Dothiorella striata*	DAR80992 *	KJ573643	KJ573640	NA
*Dothiorella symphoricarposicola*	MFLUCC 13-0498	KJ742379	KJ742382	NA
*Dothiorella symphoricarposicola*	MFLUCC 13-0497 *	KJ742378	KJ742381	NA
*Dothiorella tectonae*	MFLUCC12-0382	KM396899	KM409637	KM510357
*Dothiorella thailandica*	MFLUCC 11-0438 *	NR_111794	JX646861	JX646844
*Dothiorella thripsita*	BRIP 51876	KJ573642	KJ573639	KJ577550
*Dothiorella uruguayensis*	CBS 124908 *	NR_156208		KX464886
*Dothiorella vidmadera*	DAR 78992 *	EU768874	EU768881	HM800522
*Dothiorella vidmadera*	CBS 621.74	KX464129	KX464621	KX464887
*Dothiorella vidmadera*	CBS 725.79	KX464130	KX464622	KX464888
***Dothiorella vidmadera***	**CFCC 71191**	**PV264858**	**PV268114**	**PV339822**
***Dothiorella vidmadera***	**N282**	**PV264859**	**PV268115**	**PV339823**
*Dothiorella vinea-gemmae*	B116-3 *	KJ573644	KJ573641	KJ577552
*Dothiorella viticola*	WA10NO01 *	HM009376	HM800511	HM800519
*Dothiorella viticola*	WA10NO02	HM009377	HM800512	HM800520
*Dothiorella viticola*	IRNBS28	MN634039	MN633993	NA
*Dothiorella viticola*	MFLUCC 22-0059	ON707685	ON720571	ON677455
*Dothiorella yunnana* comb. nov.	CGMCC 3.18000 *	KX499644	KX499650	NA
***Dothiorella yunnana* comb. nov.**	**CFCC 71177**	**PV264860**	**NA**	**PV339824**
*Dothiorella zanthoxyli*	UESTCC 22.0083	OQ190537	OQ241469	OQ275070
*Dothiorella zanthoxyli*	UESTCC 22.0084	OQ190538	OQ241470	OQ275071
*Dothiorella zanthoxyli*	CGMCC 3.24159 *	OQ190536	OQ241468	OQ275069
***Dothiorella zanthoxyli***	**N280**	**PV264861**	**NA**	**PV339825**
***Dothiorella zanthoxyli***	**N280B**	**PV264862**	**NA**	**PV339826**
*Neofusicoccum luteum*	CBS 562.92 *	KX464170	KX464690	KX464968
*Neofusicoccum luteum*	CMW 41365	NR_147360	KP860702	KP860779

Note: NA, not applicable. Ex-type strains are marked with *, and strains from present study are in black bold.

**Table 3:** Isolates and GenBank accession numbers used in the genus *Phaeobotryon*.

Species	Isolates	GenBank Accession Number		
		LSU	ITS	*tef1*
*Alanphillipsia euphorbia*	CBS 136411 *	KF777196	KF777140	MT592029
*Phaeobotryon aplospora*	CFCC 53774	MN215871	MN215836	MN205996
*Phaeobotryon aplospora*	CFCC 53775 *	MN215872	MN215837	NA
*Phaeobotryon aplospora*	CFCC 53776	MN215873	MN215838	MN205997
*Phaeobotryon caraganae*	NEFU817	NA	MH014076	MH036714
*Phaeobotryon caraganae*	NEFU816	NA	MF193891	MF509765
*Phaeobotryon cupressi*	IRAN 1455C *	KX464539	FJ919672	FJ919661
*Phaeobotryon cupressi*	IRAN 1454C	KX464538	FJ919673	FJ919662
*Phaeobotryon cupressi*	IRAN 1445C	NA	KF766208	KF766428
*Phaeobotryon fraxini*	CFCC 70762 *	PP177348	PP188527	NA
*Phaeobotryon fraxini*	CFCC 70763	PP177349	PP188528	NA
*Phaeobotryon juniperi*	JU 001 *	OP941644	OP941637	OP948218
*Phaeobotryon juniperi*	JU 005	OP941645	OP941638	OP948219
*Phaeobotryon laricinum*	CFCC 70804	PP960198	PP960188	PQ046941
*Phaeobotryon laricinum*	CFCC 70805 *	PP960199	PP960189	PQ046942
*Phaeobotryon laricinum*	CFCC 70806	PP960200	PP960190	PQ046943
*Phaeobotryon longiparaphysium*	CFCC 70807 *	PP960203	PP960193	PQ046946
*Phaeobotryon longiparaphysium*	CFCC 70808	PP960204	PP960194	PQ046947
*Phaeobotryon mali*	XJAU 293001	MW367101	MW326854	MW509519
*Phaeobotryon mali*	XJAU 277201	MW367094	MW326853	MW509520
*Phaeobotryon mali*	XJAU 278201	MW367092	MW326852	MW509516
*Phaeobotryon mali*	XJAU 309401	MW367100	MW326858	MW509517
*Phaeobotryon mali*	XJAU 310001	MW367093	MW326878	MW509518
*Phaeobotryon mamane*	CPC 12442	DQ377899	EU673333	EU673299
*Phaeobotryon mamane*	CPC 12440 *	EU673248	KF766209	EU673298
*Phaeobotryon mamane*	CPC 12443	EU673249	EU673334	EU673300
*Phaeobotryon negundinis*	CAA 797	KU820971	KX061513	KX061507
*Phaeobotryon negundinis*	CAA 798	NG_069332	KX061514	KX061508
*Phaeobotryon platycladi*	CFCC 58799 *	OQ652543	OQ651172	OQ692930
*Phaeobotryon platycladi*	CFCC 58800	OQ652544	OQ651173	OQ692931
*Phaeobotryon rhoinum*	CFCC 52449	MH133940	MH133923	MH133957
*Phaeobotryon rhoinum*	CFCC 52450 *	MH133941	MH133924	MH133958
*Phaeobotryon rhoinum*	CFCC 52451	MH133942	MH133925	MH133959
*Phaeobotryon rhoinum*	XJAU 146801	MW367102	MW326857	MW509522
*Phaeobotryon rhoinum*	XJAU 276401	MW367095	MW326855	MW509524
*Phaeobotryon rhoinum*	XJAU 304901	MW367096	MW326856	MW509523
*Phaeobotryon rhoinum*	XJAU 316801	MW367097	MW326877	MW509521
*Phaeobotryon rhois*	CFCC 89662 *	KM030591	KM030584	KM030598
*Phaeobotryon rhois*	CFCC 89663	KM030592	KM030585	KM030599
*Phaeobotryon spiraeae*	CFCC 53925 *	OM049432	OM049420	NA
*Phaeobotryon spiraeae*	CFCC 53926	OM049433	OM049421	NA
*Phaeobotryon ulmi*	CBS 138854 *	MT587321	MT587540	MT592274
*Phaeobotryon ulmi*	CBS 123.30	DQ377861	KX464232	KX464766
***Phaeobotryon xizangense* sp. nov.**	CFCC 71501 *	**PV264865**	**PV264863**	**PV268116**
***Phaeobotryon xizangense* sp. nov.**	LZ204 *	**PV264866**	**PV264864**	**PV268117**
*Oblongocollomyces variabilis*	CBS 121774 *	KX464536	NR_136994	EU101357

Note: NA, not applicable. Ex-type strains are marked with *, and strains from present study are in black bold.

## Data Availability

All sequence data are available in NCBI GenBank (Table 1, Table 2 and Table 3).
